# Statistical and Machine Learning Models for Classification of Human Wear and Delivery Days in Accelerometry Data

**DOI:** 10.3390/s21082726

**Published:** 2021-04-13

**Authors:** Ryan Moore, Kristin R. Archer, Leena Choi

**Affiliations:** 1 Department of Biostatistics, Vanderbilt University Medical Center, Nashville, TN 37232, USA; ryan.moore@vumc.org; 2 Department of Orthopaedic Surgery, Center for Musculoskeletal Research, Vanderbilt University Medical Center, Nashville, TN 37232, USA; kristin.archer@vumc.org; 3 Department of Physical Medicine & Rehabilitation, Osher Center for Integrative Medicine, Vanderbilt University Medical Center, Nashville, TN 37232, USA

**Keywords:** accelerometry, statistical learning, machine learning, predictive modeling, neural networks, physical activity

## Abstract

Accelerometers are increasingly being used in biomedical research, but the analysis of accelerometry data is often complicated by both the massive size of the datasets and the collection of unwanted data from the process of delivery to study participants. Current methods for removing delivery data involve arduous manual review of dense datasets. We aimed to develop models for the classification of days in accelerometry data as activity from human wear or the delivery process. These models can be used to automate the cleaning of accelerometry datasets that are adulterated with activity from delivery. We developed statistical and machine learning models for the classification of accelerometry data in a supervised learning context using a large human activity and delivery labeled accelerometry dataset. Model performances were assessed and compared using Monte Carlo cross-validation. We found that a hybrid convolutional recurrent neural network performed best in the classification task with an F1 score of 0.960 but simpler models such as logistic regression and random forest also had excellent performance with F1 scores of 0.951 and 0.957, respectively. The best performing models and related data processing techniques are made publicly available in the R package, Physical Activity.

## 1. Introduction

The use of accelerometers has become increasingly common in engineering, industry, and consumer electronics. Due to advances in microelectronics, the proliferation of this technology has become particularly evident in healthcare research, where wearable accelerometers are often used for measuring the activity of both patients and participants in clinical trials [1]. Within the field of epidemiology, physical activity has become an important area of study and has been found to be strongly associated with health status [2] and disease progression [3]. Wearable accelerometers provide an accurate, affordable, and non-invasive method of measuring physical activity. They have been successfully utilized in a wide variety of healthcare studies such as monitoring rehabilitation following spine surgery [4], fall detection in the elderly [5], and measuring energy expenditure [6].

Although accelerometry data measured from wearable sensors provides a wealth of valuable information, the analysis of the data can present unique challenges due to massive file sizes, participant non-adherence to protocols, and the data often being collected outside of controlled laboratory settings. In epidemiological studies accelerometers are often activated prior to shipment to participants and are not deactivated until they are returned to the laboratory. This process causes large portions of the data to be recorded while the accelerometers are in transit to the participant or laboratory. Analysts must perform the arduous task of manually removing delivery activity from the dataset by looking through data logs or using data visualization techniques. As accelerometers have been increasingly used to measure physical activities in large studies (e.g., >7000 participants in Women’s Health Study [7]), manual removal of delivery activity is an especially daunting task. The purpose of this study is to develop models that can accurately classify a given day in an accelerometer dataset as “human wear” or “delivery,” and to develop an R program implementing the developed models. These models and the accompanying R program can be used to automate the removal of delivery days in accelerometry datasets before the analysis on human activity.

Our study is novel in four ways. First, to the best of our knowledge, no other models have been developed specifically for discriminating between accelerometer-measured activity from human wear and delivery. Classifying accelerometry data as human wear or delivery is traditionally performed by human labor and is very time-consuming work that can become infeasible in massive datasets. Our study is the first reporting a novel model developed to perform and automate this task. Second, we applied sophisticated neural network approaches to perform the delivery/human wear classification, which resulted in excellent performance. Third, we have provided an in-depth comparison of the performances of many different model architectures using rigorous validation techniques. Lastly, we implemented the developed neural network as well as few simpler models in an R package, which is freely available and able to function with accelerometry data from a wide variety of devices and temporal resolutions. Users can directly use our models or develop their own models with our accompanying feature extraction functions provided in the R package.

Previous studies utilizing accelerometry data adulterated with delivery activity have had to inappropriately utilize algorithms that were not developed for this specific goal or have had to perform the painstaking task of manually identifying delivery days using mail logs and visual identification of delivery days [7,8]. Algorithms have been developed for identifying intervals during which participants are not wearing an accelerometer [9,10]. These algorithms have been found to have good performance in the context of classifying wear and non-wear intervals [11,12], but the algorithms were not developed for the classification of delivery versus human activity and have had poor performance when used for this application [7].

The Women’s Health Study [7] mailed accelerometers to the study participants and tested the performance of two wear/non-wear algorithms for the classification of days in their data as either human activity or delivery. The first algorithm was used in the National Health and Nutrition Examination Survey [9] and defined non-wear time as any 60 consecutive minutes, allowing any 1 to 2 min time span with less than 100 counts per minute of activity. The second algorithm [10] was developed to improve upon the first algorithm and defined non-wear time as any 90 consecutive minutes of no activity, allowing a short time interval with nonzero counts lasting up to 2 min. The second algorithm additionally added a second window 30 min upstream and downstream during which any nonzero counts beyond the short allowed interval of movement classified the given interval as wearing. The Women’s Health Study found that wear/non-wear algorithms alone performed poorly in the context of delivery/human wear classification relative to manually labeling.

Although epidemiologic studies often mail accelerometer devices to study participants [7], the field has not developed models for discriminating between delivery and human activity. Fortunately, the field of human activity recognition is highly applicable to this classification problem and algorithms utilized to classify human activity provide a foundation for the development of models for this task. The primary goal in human activity recognition research is to classify temporal partitions in a dataset in which different activities are performed [13]. Human activity recognition is performed with data from a wide variety of sensors such as video cameras, GPS, heart monitors, thermometers, and wearable accelerometers, which are one of the most commonly utilized devices due to recent technical advances in microelectronics and the rich data they provide [14].

In the context of human activity recognition, accelerometry data are traditionally analyzed by first extracting global features such as time between peaks and average acceleration from the temporal intervals of the dataset. By extracting features, massive datasets can be reduced such that regression models or machine learning methods can be used with a reasonable number of variables. Kwapisz et al. [15] successfully utilized extracted features from accelerometer data to accurately classify six human activities with multilayer perceptron [16] and logistic regression models in the Wireless Sensor Data Mining (WISDM) project. More recently, Ellis et al. [17] developed a random forest model [18] that used extracted acceleration features to accurately classify human activity into 4 types.

Utilizing feature extraction to develop models is very common in the field of human activity recognition; however, the recent advancement of neural networks and computing power allows direct analysis of the raw data to learn complex features. The convolutional [19] and long short-term memory (LSTM) [20] neural network architectures have been used in human activity recognition due to their ability to automate the collection of local and temporal features. Ignatov [21] recently developed a convolutional neural network for the purposes of human activity recognition that learned local spatial features, while simultaneously using extracted global features. Another study [22] was successfully able to predict human activity from accelerometry data using a hybrid convolutional LSTM recurrent neural network. Recent clinical applications of convolutional recurrent neural networks used to classify human activity using accelerometry data include patient activity monitoring [23] and fall detection [24].

We considered several methods commonly used in the field of human activity recognition to develop models for the classification of days in an accelerometry dataset as human wear or delivery. Our models were developed in a supervised learning context using a large manually labeled human activity and delivery accelerometry dataset. We developed logistic regression, mixed-effects logistic regression, random forest, and multilayer perceptron models using extracted features from the dataset. Additionally, we developed convolutional neural network, LSTM recurrent neural network, and hybrid convolutional recurrent neural network models using scaled raw tri-axial accelerometer data as inputs without specific feature extraction. The models are useful for clinicians and researchers interested in classifying accelerometry data as either scientifically relevant human wear days or delivery days to be removed.

## 2. Materials and Methods

### 2.1. Data Processing

**Figure 1:**
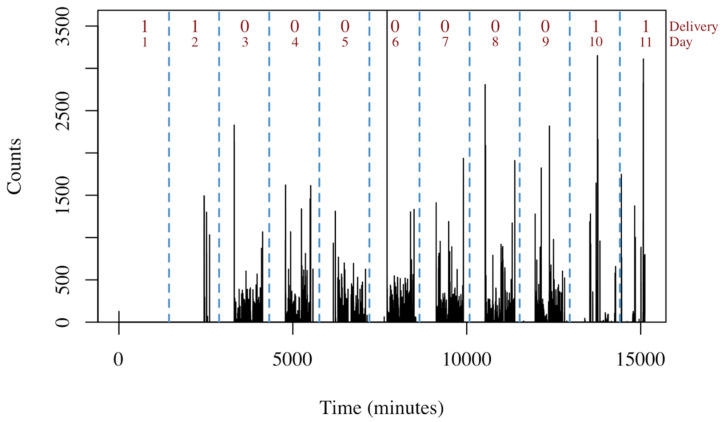
Example of accelerometry data for an assessment. The black lines represent the measurements on the *x*-axis with a one-minute epoch. Vertical dashed blue lines indicate midnight. The ‘Delivery’ label indicates 0 for a human wear day and 1 for a delivery day. The red text enumerates the day of the assessment.

The accelerometry dataset used to fit our models is composed of 779 assessments in which 251 participants were mailed a tri-axial Actigraph GT3X accelerometer (Actigraph, LLC, Pensacola, FL, USA) to wear for one week at three time-points during a randomized clinical trial in patients undergoing spine surgery. Actigraph assessments occurred at 6 weeks, 6 months, and 12 months after surgery [4,25]. Approximately 54% of the days in the dataset are delivery days, while the remainder are human wear. Physical activity was measured with 1-min epoch from the x, y, and z axes. An example of accelerometry data from the *x*-axis over the course of one assessment is shown in Figure 1.

The participants were requested to wear the Actigraph for the entire duration of the assessment, except when sleeping. Additionally, participants were requested to keep a timestamped log of when they received the Actigraph in the mail, when they returned the Actigraph to postal services, and any other potential issues such as non-adherence to protocol. These logs were used to label the days in the dataset as either human wear or delivery.

Prior to modeling, we applied two methods to process the data. As the data were measured with 1-min epoch, a complete day consists of 1440 measurements for each of the three axes. Every assessment contained at least one incomplete day during which less than 1440 measurements were taken. This occurred due to the accelerometer being activated or deactivated at any time other than midnight. Since the convolutional layers of a neural network require all inputs to be the same shape, the days that contained less than 1440 measurements were zero-padded (i.e., zero count at each minute) to a length of 1440. If the truncated day occurred at the start of the assessment, the zero-padding occurred from midnight to the time the Actigraph was activated. If the truncated day occurred at the end of the assessment, the zero-padding occurred from the deactivation time to the next midnight. Data that were only zero-padded were denoted as “minimally processed” data.

The data were also processed using procedures designed to remove days that contain little information or non-adherence. Any day was removed from the dataset if it had a total of less than 5000 counts or less than 10 min of movement in the vector magnitude of all three axes. Additionally, any day that was labeled as human wear was removed if less than 120 min of total activity occurred as this indicated a large amount of non-compliance with the protocol. The rational for this criterion is that these low activity days would likely not be included in a typical data analysis as they do not meet criteria as qualified data (e.g., many studies require 600 min of wearing to be qualified as valid day). Data that were both zero-padded and processed with aforementioned criteria were denoted as “fully processed.” The difference in processing between the two methods is summarized in Table 1.

**Figure 2:**
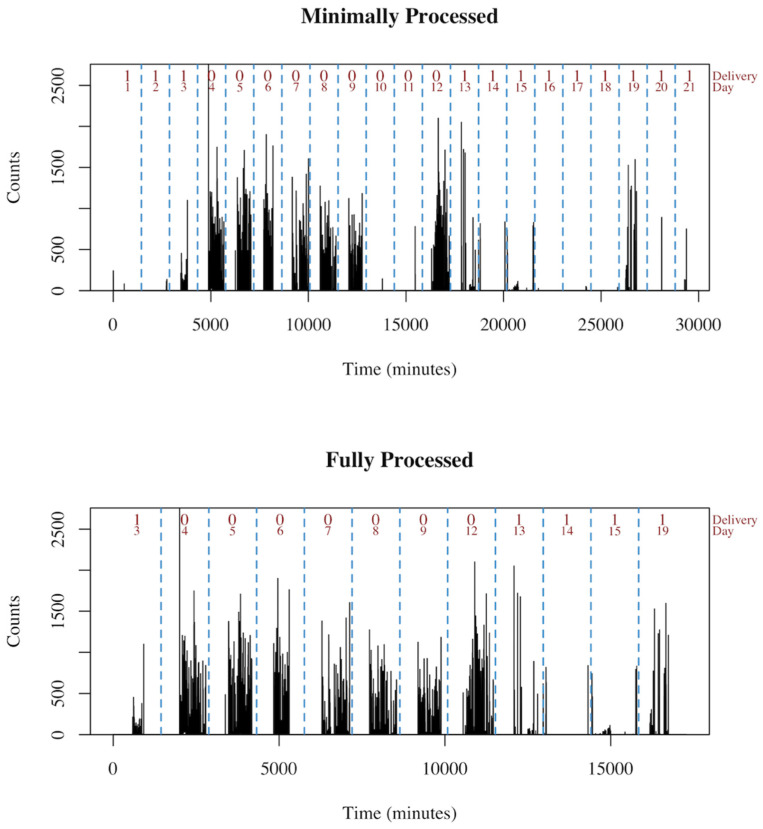
Example of minimally and fully processed data. The minimally processed data retains all of days from the raw data. The full processing removes days with non-compliant human activity (days 10–11) and delivery days with little information (days 1–2, 16–18, and 20–21).

In this analysis, both the minimally and fully processed datasets were modeled in order to explore different algorithm’s capabilities of handling messier data. The minimally processed data approximates accelerometer data that is confounded with participant non-adherence, while the fully processed data is a much cleaner dataset. An example and visualization of the differences between the minimally and fully processed data is presented in Figure 2.

The data from each day were segmented into lengths of 1440 measurements between the hours of 0:00 and 23:59. These segments were reshaped into three dimensional arrays of stacks of 1440 by 3. The day long segments were used as inputs in the convolutional and recurrent neural networks or to extract features. We extracted 8 features from the vector magnitude, which include: mean, variance, maximum, 95th quantile, absolute energy, absolute change in energy, kurtosis, and skewness. The definition of absolute energy is defined as:

(1) $$\mathrm{Absolute}\;\mathrm{Energy}=\;\overset{n}{\underset{i=1}{\sum }}x_{i}^{2}\;$$

and absolute change is defined as:

(2) $$\mathrm{Absolute}\;\mathrm{Change}=\;\sum_{i=1}^{n-1}\left|x_{i+1}-x_{i}\right|.$$

All the features and the raw data from the x, y, and z axes were mean centered and scaled by their standard deviations, which is both critical for achieving convergence in many models and helps make the models more generalizable.

### 2.2. Model Development and Validation

Seven different models were developed: random forest [18], multi-layer perceptron [16], logistic regression, mixed-effects logistic regression, convolutional neural network [19], LSTM recurrent neural network [20], and a convolutional LSTM recurrent neural network [26]. The random forest, regression models, and multi-layer perceptron utilized a traditional approach with extracted features from each data segment as inputs. On the other hand, the convolutional and recurrent neural networks used the scaled raw data as inputs in order to allow the models to learn local level features, but without including the extracted features. All neural networks were fit with a binary cross-entropy loss function and an Adam optimizer [27] over 10 epochs of training. Additional information on the neural networks’ architecture can be found in the Supplementary Materials (Tables S1–S4).

The random forest model was developed using 8 extracted features and was composed of 500 trees with a minimum terminal node size of 1. Gini impurity was utilized as the criterion for measuring the quality of each split in individual trees and the number of features considered at each split was the rounded down log base 2 of the number of total features (i.e., log_2_8 = 3) [18].

The logistic and mixed-effects logistic regression models were also fit with 8 extracted features, each of which was flexibly modeled with a three knot restricted cubic spline. The mixed effects model was fit with a random intercept for participant.

Five-fold Monte Carlo cross-validation was performed to assess model performance. For each repetition, test and training sets were selected by randomly sampling 30% and 70% of participants’ data, respectively. The models were fit with the training sets, then the mean sensitivity, positive predictive value, F1 score, and Brier score [28] were calculated from the predictions of the test sets. Sensitivity (also known as recall) is calculated as the ratio of true positive delivery classifications to true positives plus false negatives:

(3) $$\mathrm{Sensitivity}=\;\frac{\mathrm{True}\;\mathrm{Positive}}{\mathrm{True}\;\mathrm{Positive}+\mathrm{False}\;\mathrm{Negative}}.$$

Positive predictive value (PPV) (also known as precision) is calculated as the ratio of true positives to true positives plus false positives:

(4) $$\mathrm{PPV}=\;\frac{\mathrm{True}\;\mathrm{Positive}}{\mathrm{True}\;\mathrm{Positive}+\mathrm{False}\;\mathrm{Positive}}.$$

F1 score is a commonly used general measure of model performance in the field of machine learning and is calculated as the harmonic mean of sensitivity and PPV:

(5) $$F1\;\mathrm{Score}=\;\frac{2*\left(\mathrm{Sensitivity}*\;\mathrm{PPV}\right)}{\mathrm{Sensitivity}+\;\mathrm{PPV}}$$

Brier score is the mean square error of a model’s predicted probability, where f_i_ indicates a model’s forecast and o_i_ indicates the true outcome for i^th^ sample across N samples:

(6) $$\mathrm{Brier}\;\mathrm{Score}=\;\frac{1}{N}\sum_{i=1}^{N}{\left(f_{i}-o_{i}\right)}^{2}.$$

## 3. Results

### 3.1. Data Description

**Figure 3:**
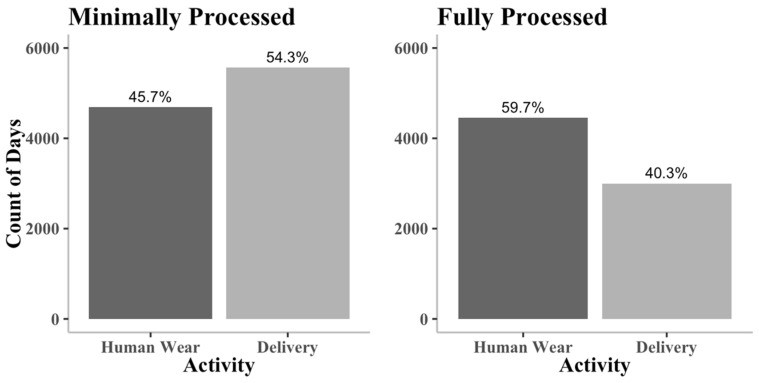
The number of days by activity in the minimally and fully processed datasets. The percentage of each activity is presented on top of each bar. The fully processed data has a lower proportion of delivery days than the minimally processed data.

After minimal processing, the data had a total of 10,546 days, while the fully processed data had a total of 7433 days. The days removed during human wear were likely caused by non-adherence. Figure 3 shows that only 46% of the days in the minimally processed dataset are human wear, while 60% of the days in the fully processed dataset are human wear. Most of the days removed from full processing are delivery days with little or no activity.

**Figure 4:**
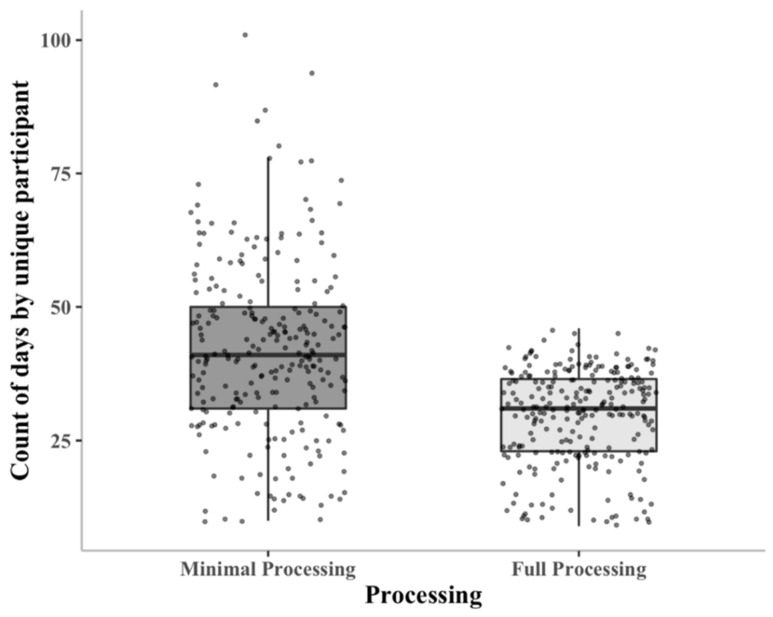
Box plots of the number of days of data per participant for the minimally and fully processed datasets. The center line of the boxplot indicates the median. The bottom and top hinges of the box indicate the 25th and 75th quantiles. The whiskers extend from the end of the box to a length of 1.5 multiplied by the interquartile range. Additionally, data points are overlaid on the boxplot.

Each subject participated in a range of one to three assessments in which they were asked to wear the Actigraph for one week. On average, the accelerometer for each assessment was active for approximately 17 days, much more than 7 days, suggesting many days are non-wear or delivery days. Across all assessments, the average number of days per participant was approximately 42 days. After fully processing the data, the average number of days per participant was reduced to approximately 30 days. The number of days by participant in the minimally and fully processed dataset is shown in Figure 4.

### 3.2. Model Performance

**Figure 5:**
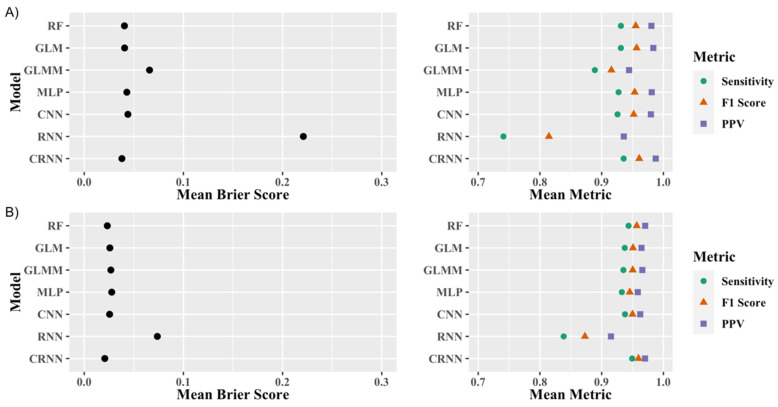
Cross-validated average model performance metrics on the (**A**) minimally processed data; (**B**) and fully processed data. The models were validated with 5-fold Monte Carlo cross-validation. RF: Random Forest; GLM: Generalized Linear Model; GLMM: Generalized Linear Mixed-Effects Model; MLP: Multilayer Perceptron; CNN: Convolutional Neural Network; RNN: Recurrent Neural Network; CRNN: Convolutional Recurrent Neural Network; PPV: Positive Predictive Value.

The mean of the sensitivity, PPV, F1 score, and Brier score across the 5 Monte Carlo cross-validations are presented in Figure 5 for the minimally (Figure 5A) and fully processed data (Figure 5B). The corresponding numerical results with both mean and standard deviation of the model performance metrics are also presented in the Supplementary Materials (Table S5). All models had a lower Brier score for the fully processed dataset compared to the minimally processed dataset; however, several models have a better F1 score in the minimally processed dataset. For both forms of processing, the recurrent architecture had the worst performance, while the convolutional recurrent neural network model marginally outperformed the other models with a mean F1 score of 0.961 and 0.960 in the minimally and fully processed datasets, respectively.

Out of the feature input models, the mixed-effects logistic regressions generally performed the worst, while the random forest marginally outperformed the other models in most of the metrics. The mixed-effect model performed fairly well when used on the fully processed dataset but performed poorly relative to the other feature input models when used to model the minimally processed data. Out of the scaled raw data input models, the recurrent neural network performed the worst. Similar to the mixed-effect model, the recurrent neural network’s performance was particularly poor relative to other models when used to model the minimally processed dataset. The convolutional neural network performed very well in both the minimally and fully processed data, but it was marginally outperformed by the convolutional recurrent neural network that performed best out of all the models.

## 4. Discussion

### 4.1. Model Performance

This study used a large dataset of 10,546 days of activity with minimal processing and 7433 days of activity after fully processing to develop models for the classification of days in accelerometry data as either human wear or delivery activity. We trained several statistical and machine learning models in a supervised learning context to discriminate between human wear and delivery days. All models performed well, especially with the fully processed data. A hybrid convolutional recurrent neural network marginally outperformed the other models with a mean 5-fold cross-validated Brier score of 0.021 and F1 score of 0.960. The logistic regression and random forest models also performed well with mean Brier scores of 0.026 and 0.023, and F1 scores of 0.951 and 0.957, respectively.

The convolutional and convolutional recurrent neural networks performed the best, while the recurrent neural network performed the worst out of all models for both the minimally processed and fully processed datasets. The slightly stronger performance of the convolutional recurrent neural network relative to the convolutional neural network indicates that incorporating time dependencies is helpful. However, the poor performance of the recurrent neural network indicates that the data greatly benefits from being reduced in dimensionality through convolutional layers before the recurrent layer processes the sequence. It is likely that the LSTM recurrent neural network has difficulties processing the thousands of days input with a length of 1440 measurements per day.

The convolutional recurrent neural network had the best performance, but the structure of the dataset makes the model somewhat naive in that it cannot differentiate between unique assessments. Ideally, the data would have been zero padded between assessments in order to reset the internal memory of the recurrent layers. Another potential improvement to the convolutional recurrent neural network would be the inclusion of a bidirectional LSTM layer. These layers incorporate information from both future and past states in an input sequence and have recently been shown to have improved performance over traditional LSTMs in certain contexts [29].

The random forest and logistic regression marginally outperformed the multilayer perceptron model. The mixed effects model performed approximately as well as the random forest and logistic regression model for the fully processed data but performed poorly when modeling the minimally processed data.

Comparing our models’ performances to a benchmark is difficult as no other models have been published specifically for predicting days as either human wear or delivery. The Women’s Health Study [7] attempted to classify days as human or delivery activity using wear/non-wear algorithms that were not developed for this specific task. They found that 27.2% to 78% of the trials were inaccurately labeled using these methods. We did not use accuracy as a metric because it is not robust in non-balanced datasets. Although their metrics are not completely comparable with ours, our F1 and Brier Scores still suggest far better performance than seen in the Women’s Study. While we cannot directly compare our model to another deliver/human-wear classification model, we can indirectly compare our models’ performances to models used in a similar context for human activity recognition. A recent study [23] investigated the use of a convolutional recurrent neural network for the classification of six unique activities commonly performed by hospitalized patients as measured by an accelerometer. Their model was validated using a 75%/25% test-train split and found to have an F1 score of 0.95. Although our models had marginally better performance relative to this human activity recognition model, it is important to note our binary classification context is simpler and easier to model than the multiclass classification commonly performed in human activity recognition research.

### 4.2. Limitations

The largest limitation of this study is that the models were developed in a supervised learning context and may not perform well with accelerometry data obtained from different studies. However, we expect reasonably good performance when the models are applied to new data considering the mechanistic nature of delivery activity and the high performance of our models during internal validation.

One major advantage for our model’s potential generalizability is its strong performance with low temporal resolution data. Many different types of accelerometers collect data at different frequencies and a model that is a trained at a high temporal resolution would be unusable for users with data collected at a lower resolution. The model was trained on a dataset with a temporal resolution of one measurement per minute (1-min epoch). Using a program such as the R package *PhysicalActivity* [30], an analyst can easily collapse any accelerometry dataset with a higher temporal resolution such as one measurement per second to our model’s 1-min epoch specification. Additionally, mean centering and standard deviation scaling of the data will likely make the models applicable to data collected from a wide variety of accelerometers other than Actigraph. While we cannot yet confirm the external validity of our models, we welcome contribution from users to validate our models in a variety of other contexts.

### 4.3. Implementation

Our models were developed in order to create a publicly available tool that can accurately automate the laborious task of classifying days in accelerometry data sets as human wear that should be further analyzed and delivery data that need to be removed prior to analysis. Currently this task can be performed by human with a high degree of accuracy; however, this task is not always feasible due to potentially massive datasets contained in thousands of files that cover years of dense activity. Although we expect our models to perform well in small datasets as well, the models would be most useful in the context of big data in which automation is vital.

Another important point to consider is the ease of each model’s implementation. The random forest and logistic regression models are fairly simple to implement on a different dataset but do require certain statistical features to be extracted. A function for extracting the features that are key to our models is available in the *PhysicalActivity* R package [30]. One advantage of the feature extraction and scaling is that the models are easily applicable to data with other temporal resolutions. The logistic regression model would be especially easy to import for use in any programming language as it has a closed form solution and would not require any package dependencies. Although the convolutional recurrent neural network showed the best performance for our dataset, it is most difficult to implement due to its dependency on the R package, *keras*. Although it is fairly simple to export and import models, the requirement of both installing *keras* and running the model could deter some users.

## 5. Conclusions

We developed several statistical and machine learning models that classify days in an accelerometry dataset as human wear or delivery activity with a high level of predictive accuracy. The majority of these models demonstrated excellent performance with both minimally and fully processed datasets. The top performing three models (random forest, logistic regression, and convolutional recurrent neural networks), feature extraction, and data processing techniques from this study are implemented in the R package, *PhysicalActivity*. Readers can find the most recent version of the R Package at https://github.com/couthcommander/PhysicalActivity (accessed on 12 April 2021). These models will allow an analyst to automate the cleaning of human activity accelerometry data that is adulterated with delivery data. In choosing the best model for application in identifying delivery days, the user can choose a model based on whether they want to use raw data or utilize manual feature extraction. The user can also weigh the higher computational cost and greater performance of the convolutional recurrent neural networks against the faster but slightly less powerful random forest or logistic regression models. Future work is needed to externally validate the models with other datasets collected in diverse studies, and we welcome contributions from users.

## Figures and Tables

**Table 1:** Summary of methods to generate minimally and fully processed data.

Minimally Processed	Fully Processed
Zeropad days with <1440 min	Zeropad days to = 1440 min
	Remove days with <5000 total counts Remove days with <120 min human activity Remove days with <10 min delivery activity

## Supplementary Materials

The following are available online at https://www.mdpi.com/article/10.3390/s21082726/s1, Table S1: Architecture of multi-layer perceptron neural network, Table S2: Architecture of 1-D convolutional neural network, Table S3: Architecture of long short-term memory (LSTM) recurrent neural network, Table S4: Architecture of convolutional long short-term memory neural network, Table S5: Average model performance metrics from 5-fold Monte Carlo cross-validation with standard deviation in parentheses for the minimally and fully processed data.

## References

[1] Lee I.-M., Shiroma E.J. (2014). Using accelerometers to measure physical activity in large-scale epidemiological studies: Issues and challenges. Br. J. Sports Med..

[2] Kohl H.W., Craig C.L., Lambert E.V., Inoue S., Alkandari J.R., Leetongin G., Kahlmeier S. (2012). Lancet Physical Activity Series Working Group. The pandemic of physical inactivity: Global action for public health. Lancet.

[3] Benzo R. (2009). Activity Monitoring in Chronic Obstructive Pulmonary Disease. J. Cardiopulm. Rehabil. Prev..

[4] Coronado R.A., Robinette P.E., Henry A.L., Pennings J.S., Haug C.M., Skolasky R.L., Riley L.H., Neuman B.J., Cheng J.S., Aaronson O.S. (2020). Bouncing back after lumbar spine surgery: Early postoperative resilience is associated with 12-month physical function, pain interference, social participation, and disability. Spine J..

[5] Bagala F., Becker C., Cappello A., Chiari L., Aminian K., Hausdorff J.M., Zijlstra W., Klenk J. (2012). Evaluation of accelerometer-based fall detection algorithms on real-world falls. PLoS ONE.

[6] Staudenmayer J., Pober D., Crouter S., Bassett D., Freedson P. (2009). An artificial neural network to estimate physical activity energy expenditure and identify physical activity type from an accelerometer. J. Appl. Physiol..

[7] Keadle S.K., Shiroma E.J., Freedson P.S., Lee I.-M. (2014). Impact of accelerometer data processing decisions on the sample size, wear time and physical activity level of a large cohort study. BMC Public Health.

[8] Van Dyck D., Cerin E., De Bourdeaudhuij I., Hinckson E., Reis R.S., Davey R., Sarmiento O.L., Mitas J., Troelsen J., MacFarlane D. (2015). International study of objectively measured physical activity and sedentary time with body mass index and obesity: IPEN adult study. Int. J. Obes..

[9] Troiano R.P., Berrigan D., Dodd K.W., Mâsse L.C., Tilert T., McDowell M. (2008). Physical activity in the United States measured by accelerometer. Med. Sci. Sports Exerc..

[10] Choi L., Liu Z., Matthews C.E., Buchowski M.S. (2011). Validation of Accelerometer Wear and Nonwear Time Classification Algorithm. Med. Sci. Sports Exerc..

[11] Choi L., Ward S.C., Schnelle J.F., Buchowski M.S. (2012). Assessment of Wear/Nonwear Time Classification Algorithms for Triaxial Accelerometer. Med. Sci. Sports Exerc..

[12] Winkler E.A.G., Paul A., Healy Genevieve N., Clark Bronwyn K., Sugiyama Takemi Matthews Charles E., Owen Neville G. (2009). Distinguishing true sedentary from accelerometer non-wearing time: Accuracy of two automated wear-time estimations. Med. Sci. Sports Exerc..

[13] Kim E., Helal S., Cook D. (2010). Human Activity Recognition and Pattern Discovery. IEEE Pervasive Comput..

[14] Lara O.D., Labrador M.A. (2013). A Survey on Human Activity Recognition using Wearable Sensors. IEEE Commun. Surv. Tutor..

[15] Kwapisz J.R., Weiss G.M., Moore S.A. (2011). Activity recognition using cell phone accelerometers. SIGKDD Explor. Newsl..

[16] Rosenblatt F. (1958). The perceptron: A probabilistic model for information storage and organization in the brain. Psychol. Rev..

[17] Ellis K., Kerr J., Godbole S., Lanckriet G., Wing D., Marshall S. (2014). A random forest classifier for the prediction of energy expenditure and type of physical activity from wrist and hip accelerometers. Physiol. Meas..

[18] Breiman L. (2001). Random Forests. Mach. Learn..

[19] LeCun Y., Haffner P., Bottou L., Bengio Y. (1999). Object Recognition with Gradient-Based Learning. Shape, Contour and Grouping in Computer Vision.

[20] Hochreiter S., Schmidhuber J. (1997). Long Short-Term Memory. Neural Comput..

[21] Ignatov A. (2018). Real-time human activity recognition from accelerometer data using Convolutional Neural Networks. Appl. Soft Comput..

[22] Ordóñez F., Roggen D. (2016). Deep Convolutional and LSTM Recurrent Neural Networks for Multimodal Wearable Activity Recognition. Sensors.

[23] Fridriksdottir E., Bonomi A.G. (2020). Accelerometer-Based Human Activity Recognition for Patient Monitoring Using a Deep Neural Network. Sensors.

[24] Santos G.L., Endo P.T., de Monteiro K.H.C., da Rocha E.S., Silva I., Lynn T. (2019). Accelerometer-Based Human Fall Detection Using Convolutional Neural Networks. Sensors.

[25] Archer K.R., Haug C.M., Pennings J. (2020). Combining Two Programs to Improve Disability, Pain, and Health Among Patients Who Have Had Back Surgery.

[26] Shi X., Chen Z., Wang H., Yeung D.-Y., Wong W., Woo W.C. (2015). Convolutional LSTM Network: A Machine Learning Approach for Precipitation Nowcasting. arXiv.

[27] Kingma D., Ba J. (2014). Adam: A method for stochastic optimization. arXiv.

[28] Brier G.W. (1950). Verification of Forecasts Expressed in Terms of Probability. Mon. Weather Rev..

[29] Chiu J.P.C., Nichols E. (2016). Named Entity Recognition with Bidirectional LSTM-CNNs. Trans. Assoc. Comput. Linguist..

[30] Choi L., Beck C., Liu Z., Matthews C.E., Buchowski M.S. (2021). Physical Activity Process Accelerometer Data for Physical Activity Measurement. R Package Version 0.2-4. https://CRAN.R-project.org/package=PhysicalActivity.

