# Supplementary material for: Statistical and Machine Learning Models for Classification of Human Wear and Delivery Days in Accelerometry Data
Source: Sensors (Basel). 2021 Apr 13;21(8):2726. doi: 10.3390/s21082726 (PMC8069625; doi:10.3390/s21082726)
Supplement: Supplementary file 1 [file sensors-21-02726-s001.pdf]

sensors

MDPI

Article

Statistical and Machine Learning Models for Classification of Human Wear and Delivery Days in Accelerometry Data

Ryan Moore [1], Kristin R. Archer [2,3] and Leena Choi [1,*]

[1] Department of Biostatistics, Vanderbilt University Medical Center, Nashville, TN 37232, USA; ryan.moore@vumc.org
[2] Department of Orthopaedic Surgery, Center for Musculoskeletal Research, Vanderbilt University Medical Center, Nashville, TN 37232, USA; kristin.archer@vumc.org
[3] Department of Physical Medicine & Rehabilitation, Osher Center for Integrative Medicine, Vanderbilt University Medical Center, Nashville, TN 37232, USA
* Correspondence: leena.choi@vumc.org; Tel.: +1-615-343-3497

Citation: Moore, R.; Archer, K.R..; Choi, L. Statistical and Machine Learning Models for Classification of Human Wear and Delivery Days in Accelerometry Data. *Sensors* **2021**, *21*, 272. https://doi.org/10.3390/s21082726

Academic Editor: Ki H. Chon

Received: 12 March 2021
Accepted: 9 April 2021
Published: 13 April 2021

Publisher's Note: MDPI stays neutral with regard to jurisdictional claims in published maps and institutional affiliations.

Copyright: © 2021 by the authors. Submitted for possible open access publication under the terms and conditions of the Creative Commons Attribution (CC BY) license (http://creativecommons.org/licenses/by/4.0/).

Table S1. Architecture of multi-layer perceptron neural network.

Layer	Layer Hyperparameters	Activation Function	Output Shape
Dense	200 Neurons	ReLU	(,200)
Dropout	50% Dropout Rate		(,200)
Dense Output	1 Neuron	Sigmoid	(,1)

Table S2. Architecture of 1-D convolutional neural network.

Layer	Layer Hyperparameters	Activation Function	Output Shape
LSTM	30 Neurons	TanH and Sigmoid	(,30)
Dropout	40% Dropout Rate		(,30)
Dense	100 Neurons	ReLU	(,100)
Dropout	30% Dropout Rate		(,100)
Dense Output	1 Neuron	Sigmoid	(,1)

Table S3. Architecture of long short-term memory (LSTM) recurrent neural network.

Layer	Layer Hyperparameters	Activation Function	Output Shape
Convolutional	200 5 × 3 Filters	ReLU	(,1431,200)
Max Pooling	4 Unit Pool Size		(,357,200)
Convolutional	64 5 × 1 Filters	ReLU	(,355,64)
LSTM	30 Neurons	TanH and Sigmoid	(,30)
Dropout	30% Dropout Rate		(,30)
Dense Output	1 Neuron	Sigmoid	(,1)

Table S4. Architecture of convolutional long short-term memory (LSTM) neural network.

Model	Sensitivity	PPV	F1 Score	Brier Score
Minimally Processed Dataset				
Feature Input Models				
Random Forest	0.981 (0.008)	0.931 (0.006)	0.955 (0.006)	0.041 (0.004)
Generalized Linear Model	0.984 (0.002)	0.931 (0.004)	0.957 (0.002)	0.041 (0.002)
Generalized Mixed Effects Model	0.944 (0.040)	0.889 (0.033)	0.916 (0.036)	0.066 (0.019)
Multilayer Perceptron	0.981 (0.004)	0.927 (0.004)	0.953 (0.004)	0.043 (0.003)
Scaled Data Input Models				
Convolutional Neural Network	0.980 (0.003)	0.926 (0.012)	0.952 (0.007)	0.044 (0.008)
Recurrent Neural Network	0.936 (0.055)	0.741 (0.163)	0.815 (0.090)	0.221 (0.190)
Convolutional Recurrent Neural Network	0.988 (0.005)	0.936 (0.006)	0.961 (0.005)	0.038 (0.004)

Table S5. Average model performance metrics from 5-fold Monte Carlo cross-validation with standard deviation in parentheses for the minimally and fully processed data. PPV: Positive Predictive Value.

Fully Processed Dataset				
Feature Input Models				
Random Forest	0.970 (0.007)	0.944 (0.004)	0.957 (0.005)	0.023 (0.001)
Generalized Linear Model	0.964 (0.005)	0.937 (0.006)	0.951 (0.005)	0.026 (0.003)
Generalized Mixed Effects Model	0.966 (0.007)	0.935 (0.008)	0.950 (0.006)	0.027 (0.004)
Multilayer Perceptron	0.958 (0.005)	0.933 (0.005)	0.945 (0.005)	0.028 (0.002)
Scaled Data Input Models				
Convolutional Neural Network	0.962 (0.015)	0.938 (0.009)	0.950 (0.010)	0.026 (0.004)
Recurrent Neural Network	0.915 (0.047)	0.839 (0.068)	0.873 (0.035)	0.074 (0.040)
Convolutional Recurrent Neural Network	0.970 (0.008)	0.949 (0.006)	0.960 (0.006)	0.021 (0.002)
